# Establishing a Mental Health Surveillance in Germany: Development of a framework concept and indicator set

**DOI:** 10.25646/8861

**Published:** 2021-12-08

**Authors:** Julia Thom, Elvira Mauz, Diana Peitz, Christina Kersjes, Marion Aichberger, Harald Baumeister, Anke Bramesfeld, Jurand Daszkowski, Theresa Eichhorn, Wolfgang Gaebel, Martin Härter, Frank Jacobi, Joseph Kuhn, Jutta Lindert, Jürgen Margraf, Hanne Melchior, Andreas Meyer-Lindenberg, Angelika Nebe, Heather Orpana, Judith Peth, Ulrich Reininghaus, Steffi Riedel-Heller, Uwe Rose, Georg Schomerus, Daniela Schuler, Ursula von Rüden, Heike Hölling

**Affiliations:** 1 Robert Koch Institute, Berlin, Department of Epidemiology and Health Monitoring; 2 Department of Psychiatry and Psychotherapy at the Charité Campus Mitte, Charité – Universitätsmedizin Berlin; 3 University of Ulm, Institute of Psychology and Education, Department of Clinical Psychology and Psychotherapy; 4 Ministry for Social Affairs, Health and Equal Opportunities of Lower Saxony; 5 Hannover Medical School (MHH), Institute for Epidemiology, Social Medicine and Health System Research; 6 Federal Association of Psychiatry Experienced; 7 Federal Chamber of Psychotherapists in Germany; 8 WHO Collaborating Centre DEU-131; Rhineland Regional Council (LVR) – Klinikum Düsseldorf, Kliniken der Heinrich-Heine-Universität Düsseldorf; 9 University Medical Center Hamburg-Eppendorf, Center for Psychosocial Medicine, Department of Medical Psychology; 10 German Network Health Services Research; 11 Psychologische Hochschule Berlin, Department of Clinical Psychology and Psychotherapy; 12 Bavarian Health and Food Safety Authority; 13 University of Applied Sciences Emden/Leer; 14 European Public Health Association, Section Public Mental Health; 15 Ruhr-University Bochum, Mental Health Research and Treatment Center; 16 National Association of Statutory Health Insurance Physicians; 17 Central Institute of Mental Health, Medical Faculty Mannheim, Universität Heidelberg; 18 German Association for Psychiatry, Psychotherapy and Psychosomatics e.V.; 19 Federation of German Pension Insurance Institutions; 20 Public Health Agency Canada; 21 University of Leipzig, Faculty of Medicine, Institute of Social Medicine, Occupational Health and Public Health; 22 Federal Institute for Occupational Safety and Health; 23 University of Leipzig, Medical Faculty, University of Leipzig Medical Center, Department of Psychiatry and Psychotherapy; 24 Swiss Health Observatory (OBSAN); 25 Federal Centre for Health Education

**Keywords:** PUBLIC HEALTH, SURVEILLANCE, MENTAL HEALTH, MENTAL DISORDERS, INDICATOR

## Abstract

In the course of the recognition of mental health as an essential component of population health, the Robert Koch Institute has begun developing a Mental Health Surveillance (MHS) system for Germany. MHS aims to continuously report data for relevant mental health indicators, thus creating a basis for evidence-based planning and evaluation of public health measures. In order to develop a set of indicators for the adult population, potential indicators were identified through a systematic literature review and selected in a consensus process by international and national experts and stakeholders. The final set comprises 60 indicators which, together, represent a multidimensional public health framework for mental health across four fields of action. For the fifth field of action ‘Mental health promotion and prevention’ indicators still need to be developed. The methodology piloted proved to be practicable. Strengths and limitations will be discussed regarding the search and definition of indicators, the scope of the indicator set as well as the participatory decision-making process. Next steps in setting up the MHS will be the operationalisation of the single indicators and their extension to also cover children and adolescents. Given assured data availability, the MHS will contribute to broadening our knowledge on population mental health, supporting a targeted promotion of mental health and reducing the disease burden in persons with mental disorders.

## 1. Introduction

In recent years, the international public health agenda in the field of non-communicable diseases (NCDs) has undergone significant change. While the focus has long been on physical diseases such as cancer, diabetes, cardiovascular diseases and chronic respiratory diseases, since 2018 the World Health Organization (WHO) has also attached the highest importance to mental disorders and well-being for population health [1]. In 2015, the United Nations declared the promotion of mental health and well-being and the strengthening of approaches to prevent and treat substance abuse as explicit policy goals for sustainable development [2]. However, many countries still lack the necessary reporting systems for a robust data-based assessment of health developments in the comparatively emergent field of public mental health. Accordingly, the WHO Mental Health Action Plan (2013–2020, which has been extended until 2030) emphasises the need to establish national information systems for mental health indicators as one of its four objectives [3].

Public Health is defined as ‘the art and science of preventing disease, prolonging life and promoting health through the organized efforts of society‘ [4, as cited in 5]. Public Mental Health applies the concept of public health to mental health and disorders [6]. Surveillance in the field of public health refers to the ongoing and systematic collection, collation, analysis, interpretation and timely dissemination of data on health and well-being and their determinants [7]. Surveillance thus serves as a basis for planning, implementing and evaluating measures to protect and promote health in the population. The approach is centred around a defined set of meaningful and reliably measurable indicators (Info box 1). These are populated with data which is collected and reported continuously and can be compared over time to identify changes as well as specific needs for different population groups (stratified by age, gender, education, etc.).

In contrast with physical non-communicable as well as infectious diseases, Mental Health Surveillance (MHS) as a continuous and indicator-based reporting of mental health poses specific challenges: (1) mental health is a broad concept and as such encompasses more than merely the absence of mental disorders [9]. The dual-factor model separates mental health into two interrelated, but distinct dimensions of positive mental health (well-being) and psychopathology (symptoms and disorders) [10, 11]. In terms of their epidemiology, development, course and disease burden, the group of mental health conditions includes a highly diverse set of disorders. Moreover, despite suffering from psychopathology, people can experience their mental health as positive [11] because mental health describes ‘a state of wellbeing, in which an individual realizes his or her own abilities, can cope with the normal stresses of life, can work productively and is able to make a contribution to his or her community’ [12]. Such an approach to mapping mental health in its entirety therefore resembles the attempt to develop a set of indicators to describe ‘physical health’ (instead of ‘physical illnesses’). For physical NCDs, this is not yet commonly done, as there are usually separate surveillance subsystems for each respective disease and its risk factors that do not include salutogenetic (health-related) determinants and outcomes [13]. In addition, the interaction of risk and protective factors with mental health and the outcomes of mental disorders is extremely complex [14]. For many topics, there are age- and culture-specific constructs. Accordingly, public health-oriented reviews of potential MHS indicators produce broad results [15, 16] with a respective requirement for selection and prioritisation. (2) Unlike the laboratory parameters in the case of physical illnesses, the indicators for mental health are rarely directly observable or discretely measurable. Thus, a population-based, valid and reliable measurement of mental health poses high demands on data collection [17, 18]. Many constructs lack a gold standard for their recording in health surveys. At the same time, partly due to advancements in research, the classification of mental disorders changes, i.e. regarding their definition and the classification of specific clinical pictures, their diagnostic criteria and threshold values [19]. This complicates the establishment of standards for surveillance and can, in some cases, require a dynamic adjustment of disorder categories. Determining the prevalence of mental disorders in the population requires in-depth and hence resource-intensive clinical interviews. In addition, different data sources (survey vs routine data) may provide different estimates for one construct and then require triangulation (i.e. a comparative discussion) [20]. (3) Furthermore, in the case of mental disorders, social stigma constitutes a special source of bias that influences data collection in self-report and observer rating and can also lead to misclassifications in health care settings [17].

Systems of Mental Health Surveillance developed to date have adopted different approaches to deal with these requirements. As the following international examples illustrate, they differ considerably in terms of content focus and degree of implementation. In some cases (e.g. Switzerland [21, 22] and Australia [23]), selected indicators of mental health are recorded and communicated in various reporting formats (health reports, interactive websites or dashboards, reports for the evaluation of political objectives, etc.) yet without explicitly setting up a systematic surveillance system. In Canada, three independent subsystems within a comprehensive system of public health surveillance regularly provide information on positive mental health (well-being) [24] and on suicidality [25], taking the respective risk and protective factors into account, as well as on mental disorders diagnosed in health care settings (Canadian Chronic Disease Indicators) [26]. In the US, a set of indicators focuses on mental disorder prevalence and care, with particular emphasis on substance abuse and this is currently being tested by several states [27]. The Scottish indicator set on well-being and mental health has only once reported results on indicators of positive mental health (well-being), psychopathology and their determinants [28] notwithstanding additional quality assurance indicators developed for the care sector [29]. Such systematic quality monitoring systems focussed on the provision of care for people with mental disorders on the basis of routine data has been implemented or is currently being introduced by several countries [30, 31], meaning that these systems are currently more developed than monitoring with an epidemiological or public health focus. Additionally to the aforementioned differences in individual countries’ surveillance systems, different methods were used to develop and select indicators. For instance, either clearly operationalised measures [29, 32] or theoretical constructs without a definition of numerator and denominator [15, 24, 33] included as indicators in the development process. Research and selection of indicators may be limited to those with available data [32, 34] or may also include constructs that can be captured in principle but for which no data are currently available [16, 23, 24, 33]. Key decisions in indicator system set-up can be made either by health monitoring professionals [29, 34] or through the involvement of various stakeholders [16, 24, 32, 33]. All methodologies have their specific advantages and disadvantages for the resulting surveillance system with regard to e.g. feasibility and acceptance as well as their capacity to deal with data gaps.

In addition to meeting a country’s information needs, an MHS system should ideally also serve the country’s international reporting obligations. These include, for example, the WHO Mental Health Atlas [35] regularly requests cross-national data to assess the achievement of the Mental Health Action Plan [3]. International comparability is also the focus of the Organisation for Economic Co-operation and Development’s (OECD [36]) Mental Health Performance Benchmark, for which indicators and reporting formats are currently being developed. At EU level, after initial preliminary work to develop a cross-national indicator set on mental health [15, 16], only a few parameters have been bindingly included in the 88 European Health Indicators to date, and databases have not yet been generated for most of them [37]. In principle, the realisation of international comparative reporting by a national MHS will also depend on the extent to which these indicators are also meaningful at country level.

**Info box 1 What is an indicator?**

An indicator is a precisely defined measure which is used to describe an underlying construct (indicandum) as comprehensively as possible. It consists of the ‘metadata’ (name of the indicator and definition for quantification, e.g. its numerator and denominator concept) as well as the data itself [8].

For comprehensive public health surveillance, indicators should represent all relevant fields of action of public health measures (health promotion, prevention, treatment and rehabilitation) as well as a dimensional spectrum of health and its determinants.

### 1.1 Public Mental Health reporting in Germany

A concise overview of central developments of public mental health in Germany is only possible to a limited extent and contains gaps. Although large amounts of data on the mental health of the population are available from both studies and health care, the diversity of its contents, collection purposes and data holders provides an overall fragmented data situation. Furthermore, health policy measures for the care and prevention of mental disorders in Germany are characterised by the country’s federal structure and the responsibility of numerous ministries and actors, in addition to which such measures are generally organised across several professional groups, sectors and cost bearers [38]. Thus, it is hard to provide findings on overaching developments.

Consequently, reviews of the population’s mental health and mental disorders usually come with an array of indicators and this heterogeneity thwarts a consistent summary interpretation and discussion [39–46]. The data sources included are multiple kinds of raw data in different reporting formats, such as psychiatry reports and studies conducted by the federal states [e.g. 47, 48, 49] focus reports by individual health insurers [50–53] and other service providers and support systems [54, 55], expert reports from various research institutions [56, 57] as well as results from the nationwide health monitoring at the Robert Koch Institute (RKI) [e.g. 58] and other population studies [59]. Indicators differ in terms of case definition, operationalisation, the underlying sample or reference population as well as survey design and mode. Incongruent observation periods and a lack of longitudinal studies make it particularly difficult to assess trends and interactions of developments in morbidity and health care cannot be mapped validly. Another factor contributing to this situation is that, for most studies, only a few measurement points are available, e.g. to determine the prevalence of mental disorders in the general population by a standardised diagnostic interview for example (last recorded with the survey period 2009–2012 [60]).

Furthermore, reporting concerning some aspects of public mental health presents gaps that are owed as much to a lack of research and indicator development on individual topics, as to the lack of population-based data sources in various areas.

Moreover, the data situation can be expected to improve for indicators on mental health quality of care. However, since a cross-sectoral and cross-disorder quality assurance procedure (QA procedure) could not be implemented [61], even after the implementation of the indicator-based QA procedures currently being developed for schizophrenia, schizotypal and delusional disorders [62] and for outpatient psychotherapy [63], information will only be available on diagnosis- or therapy-specific subgroups of patients. In addition, eight quality indicators on the provision of care to people with unipolar depression are integrated in the Federal Joint Committee’s Disease Management Programme (DMP) Depression [53]. Following implementation of the DMP in care across Germany a nationwide uniform recording of these indicators will become available. Consequently, a MHS should also provide indicators of quality of care, and these, depending on the development stage of QA procedures and DMP implementation, could also be included in the selection of indicators.

It is worth noting that, from the point of view of quality monitoring, there is an explicit demand for its parameters to be evaluated in conjunction with epidemiological measures continuously being collected, as the public health impact can only be recognised in this way as an overall effect of the health care system on population health [31] and developments in care provision thus can be interpreted against the background of changing needs [64].

In summary, a consensus on key public mental health parameters and a systematic and continuous data provision still need to be established. Otherwise it is difficult to assess the extent to which health policy goals are being met and whether health care and public health measures are having an effective impact on population health. In 2009, while evaluating the health goal depression, a clear but unmet need to generate and develop meaningful data sources [65] was recognised. This criticism that there was still no or only insufficient information available on key aspects was voiced again in 2018 [66].

### 1.2 Setting up Mental Health Surveillance at the Robert Koch Institute

For a long time, in Germany too, the surveillance approach has been limited to infectious diseases and cancer. A concept for NCD surveillance is currently being developed at the RKI and has already been successfully introduced for diabetes since 2016 [67]. Following this example, the Federal Ministry of Health commissioned the RKI to start developing a Mental Health Surveillance (MHS) for Germany in 2019. The current pilot phase is focused on designing and testing the systematic development of a set of public mental health indicators. This phase should yield findings on the current state of research as well as research needs for individual indicators and therefore lay the ground for their future integration into the planned NCD surveillance. The project is initially limited to parameters for the adult population. This article describes and discusses the systematic development of a set of indicators as part of the piloting of an MHS for Germany.

## 2. Methodology

**Figure 1:**
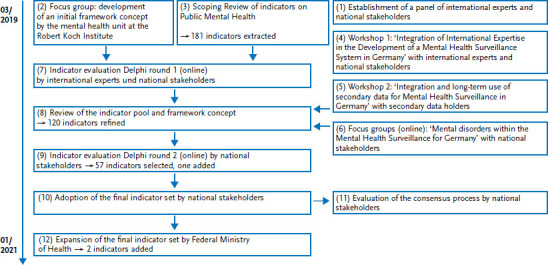
Process of development: Indicator set and framework concept Source: Own figure

In order to facilitate a later integration into a superordinate NCD surveillance based on uniform procedures, the conception of the MHS indicator set used the tried and tested method of the Diabetes Surveillance at the Robert Koch Institute as a blueprint [67]. WHO’s recommendations for the establishment of a mental health information system were taken into account [68]. Figure 1 provides an overview of the steps in this process and is the reference for the ordinals (1) – (12) used below.

One key methodological decision needs to be pointed out in advance: The term indicator was limited to the indexed theoretical construct without a specific operationalisation for its quantification with defined numerator and denominator [8]. Since each construct to be agreed upon contains various options for operationalisation depending on precise definition, measurement and data basis, this pragmatic simplification should facilitate the summarised presentation and comparative evaluation of the extraordinarily large number of potential indicators in the course of the selection process. In addition, the further process of setting up the MHS can benefit from high flexibility when clarifying hitherto not specified data access.

### 2.1 Involvement of international expertise and national stakeholders

#### Participation by experts and stakeholders (1)

For a surveillance system to effectively protect and promote public health, it must serve the specific information needs of citizens, decision-makers and researchers. Quality, a successful implementation also on the long-term, as well as acceptance and use of the system benefit from the participation of relevant actors with their respective expertise and interests. For this reason, a diverse panel of public mental health stakeholders was invited to participate (Annex Table 1). 29 representatives of national stakeholders from science, service providers, patient organisations, federal and state politics as well as federal departmental research institutions took part. In order to strengthen international comparability and to learn from experiences made in other countries, experts from the WHO Mental Health Atlas and the OECD Mental Health Performance Benchmark as well as from two public health institutes with systematic mental health reporting (Swiss Health Observatory, Public Health Agency Canada) could be won over for the project.

#### Workshops (4, 5) and focus groups (6)

A two-day workshop served as a kickoff to the process of opinion-forming by national stakeholders and the integration of international expertise in MHS development for Germany [69]. At a one-day workshop, representatives from various holders of routine data (Central Institute for Statutory Health Insurance Physicians in Germany, German Institute for Medical Documentation and Information, Scientific Institute of the AOK, Research Data Centre of the German Pension Insurance) as well as a representative of the Epidemiological Survey of Substance Abuse (Institute for Therapy Research) presented the potential the respective data bases have for quantifying public mental health indicators. The findings were incorporated into the description of potential indicators in Delphi round 2(9).

Due to the COVID-19 pandemic, the third face-to-face workshop was changed to a set of four online focus groups [70]. Topics of discussion with national stakeholders included the methodologies for selecting those mental disorders with the highest public health relevance and the evaluation of their assessment in surveys and routine data. The discussion results were used to refine Delphi Round 2(9).

#### Quantitative surveys (7, 9)

Two online surveys were conducted as part of the Delphi process (7, 9). The technical implementation was carried out based on the VOXCO software, the evaluation of the data was carried out using MS Excel 2019. The surveys were subject to data protection regulations under the Federal Data Protection Act and they were vetted and approved from a data protection perspective.

### 2.2 Development of potential indicators and framework concept

#### Focus group to develop an initial framework concept (2)

A framework concept aims at the classification of indicators according to content within an overarching, coherent, scientifically based model and can guide action [71]. To ensure compatibility with the preliminary work on NCD surveillance, the framework concept of Diabetes Surveillance at the RKI was used as a starting point [67] and further developed for the field of Public Mental Health by a focus group of researchers at the RKI’s Mental Health Unit (Department of Epidemiology and Health Monitoring).

#### Scoping review to identify potential indicators (3)

An extensive literature review of relevant public mental health indicators was conducted in the form of a scoping review [72, 73] to identify potential indicators for MHS. Over and above determining indicators which had already been established, the aim was also to identify new indicators not yet established in reporting systems in terms of content or methodology. To gain an as complete picture as possible and to include various sources of information, a systematic MEDLINE search via PubMed (limited to German and English language publications) was supplemented by additional searches on the websites of health care actors in Germany, public health institutes of all OECD (Organisation for Economic Co-operation and Development) member states, as well as major international organisations (WHO, OECD, EU). A detailed description of the research process can be found elsewhere [74].

**Info box 2 What is a Delphi process?**

The Delphi method is an iterative process in which group opinions on an issue can be obtained through repeated questioning and feedback [77]. The agreement (consensus) or disagreement (dissent) of opinions can be recorded and thus a voting result with the highest possible level of agreement sought. The procedure can be used in an anonymous format using a written survey and is widely established in the development of indicator systems [16, 24, 32, 75, 76].

### 2.3 Consensus on the final indicator set and framework concept

#### Indicator evaluation Delphi round 1 (online) (7)

The panel of international experts and national stakeholders subsequently evaluated the identified indicators via a two-stage Delphi process (Info box 2). Both Delphi rounds were conducted as online surveys. For Delphi round 1, the indicators that were identified in the scoping review were illustrated through examples of their operationalisation based on the researched sources. Each indicator was individually assessed regarding its relevance based on a nine-point scale. Relevance was defined in line with the criteria below [24] which have also been used in the development of other public and mental health indicator systems [15, 23, 75, 76]:

► Significant: the indicator has the potential to improve the protection and promotion of population mental health
► Actionable: the indicator provides information to update, influence or change policy and public health practice and can itself be influenced by policy and public health practice

A comment field was used to request comments on the individual indicators (e.g. reasons for the assessment made, alternative operationalisations, notes on options to merge the indicator with other indicators or splitting of the indicators into various sub-indicators).

To evaluate the survey, the indicators were then split into four groups based on the respondents’ assessment of their relevance [cf. 67].

► highly relevant: ≥75% of the ratings with 7 to 9 points
► relevant: ≥50%-74% of the ratings with 7 to 9 points
► medium relevant: ≥50% of the ratings with 1 to 6 points
► low relevant: ≥50% of the ratings with 1 to 3 points

The quantitative result of Delphi round 1 was fed back to the respondents.

#### Review of the framework concept and indicators (8)

Based on the qualitative feedback provided via the comment fields in the survey, adjustments were made to the framework concept (renaming of fields of action and definition of topics) as well as to the indicators (mergers, deletions and additions). Additional literature reviews were conducted on selected topics (e.g. mental health promotion and prevention) and thematically focused discussions with additional experts were carried out.

#### Indicator evaluation Delphi round 2 (online) (9)

During Delphi round 2, indicators were selected for given topics within each field of action to achieve a balanced indicator set in terms of content [cf. 76]. It was determined, that each topic had to have at least two indicators assigned to it in order for it to be adequately represented [cf. 76]. In addition, in order to reduce the number of indicators, a survey format was used that required respondents to prioritise between indicators [cf. 24]. The respondents were asked to rank how aptly an indicator represented the respective topic compared to the others (Annex Table 2). Information on (possible) operationalisations and data sources for indicators was provided.

To prioritise how compatible the final indicator set was with the information needs and data availability in Germany only the German stakeholders could take part in the online survey. The survey was divided into two parts in order to reduce the amount of time required for each survey.

The aim of the evaluation, which was communicated in advance, was to reduce the number of indicators by at least 50% in favour of a more manageable set [cf. 24] and to consider a measure of agreement among respondents [78]. Accordingly, two criteria were defined for assessing the relevance of indicators:

► Ranking (cumulated): an indicator is relevant if it belongs to the indicators with a ranking above the 50% mark across all made assessments
► Consensus: an indicator is relevant if more than 50% of the respondents ranked it above the 50% mark

The indicators were classified according to these criteria as follows:

► Highly relevant: both criteria (rank and consensus) met
► Relevant: only one of the criteria (rank or consensus) met
► Not relevant: neither of the criteria met

Both highly relevant and relevant indicators were included in the final MHS indicator set.

#### Adoption of the indicator set (10)

To capture stakeholders’ approval of the voting result of Delphi round 2 (Annex Table 2) as well as the resulting indicator set, both were sent to them by e-mail with the request to adopt or reject it as the final project result.

#### Evaluation of the consensus process (11)

To evaluate the consensus-finding process, national stakeholders were asked for a standardised assessment as part of another anonymous online survey (regarding participation, agreement with the final project result, transparency of the procedure, workload, and personal willingness for further participation).

#### Expansion of the final indicator set (12)

Public health surveillance must be orientated towards health policy information needs so it can fulfil its role in the governance of measures according to the definition of surveillance. The Federal Ministry of Health (German: Bundesministerium für Gesundheit, BMG), as the commissioner and promoter of the development of MHS, did not participate in Delphi rounds 1 and 2, as the evaluation of indicators was to remain in the hands of national and international experts and stakeholders. The BMG reserved the right to review and, if necessary, expand (but not reduce) the approved set of indicators to add relevant indicators for health policy not reflected in the set agreed upon by the experts if necessary. In this way, the participation and positioning of the BMG could be communicated transparently.

In summary, the tasks were distributed as follows among the parties involved: the MHS working group at the RKI searched indicators (Scoping Review, 3) and, together with other mental health experts at the RKI, developed an initial system to structure them (initial framework, 2); invited international experts and national stakeholders to participate in the panel (1); organised and moderated a dialogue among experts in the form of workshops and focus groups (4, 5, 6); conducted three survey studies on indicator assessment (7, 9) and evaluation (11), evaluated these based on own methodology and reported results back to the participants; revised the indicator set and framework concept on the basis of the results (8) and obtained votes on the adoption of the indicator set (10) and its expansion (12). The experts and stakeholders involved consequently contributed their expertise at the joint events (4, 6), evaluated the indicators in the two Delphi surveys (7, 9), adopted the indicator set (10) and assessed the work process during the course of the evaluation (11).

## 3. Results

### 3.1 Development of potential indicators and framework concept

#### Development of an initial framework concept (2)

The focus group at the RKI identified 13 central topics in the field of public mental health and subsequently assigned them to five superordinate fields of action following the framework concept of the Diabetes Surveillance [67, 74]. They consisted of (1) Promoting mental well-being of the population: mental health promotion and prevention, mental health resources, positive mental health (well-being); (2) Reducing the risks of mental disorders: risk factors, psychopathology, self-harm and suicidality; (3) Improving mental health care: supply and utilisation, needs, unmet needs and barriers, quality of care; (4) Reducing the burden of disease and improving participation: costs, burden of disease, participation; (5) Strengthening knowledge and acceptance: mental health literacy. In addition, sociodemographic influencing characteristics were included as an individual field.

#### Scoping review to identify potential indicators (2)

13,811 publications were identified using various research strategies. 373 of them were used to extract a total of 1,505 relevant indicators. These were categorised accordingly, deduped and assessed for compatibility (how they adapt to the care structures in Germany, indicators for adult age, etc.). In total, 181 indicators of Public Mental Health could be identified, of which 47 (26%) were not included in any national and international indicator system. An additional eleven socio-demographic characteristics affecting mental health were not assigned to any field of action, but were included as potential stratification variables to identify particularly burdened population groups in a MHS context. Details of data extraction and processing (overview of the identified indicators) are presented elsewhere [74]. The number of indicators identified per topic and field of action was not equally distributed (Table 1), meaning that the topic area ‘mental health promotion and prevention’ was underrepresented compared to the topic area ‘supply and utilisation’ for example. Eight indicators were combined because their constructs overlapped; thus, Delphi round 1 started with 173 indicators and 11 stratification characteristics.

### 3.2 Consensus on the final indicator set and framework concept

#### Indicator evaluation Delphi round 1 (online) (7)

A total of 22 fully completed data sets were included in the evaluation (response rate: 91.7%). Of the 173 indicators, 35.3% were assessed as highly relevant, 48.0% as relevant, 15.6% as medium relevant and 1.2% as low relevant (Table 1). Of the stratification characteristics, 72.7% were assessed as highly relevant and 27.3% as relevant.

A majority of the indicators in fields of action 2 to 5 as well as the socio-demographic stratification characteristics were rated as highly relevant and relevant. Only in field of action 1 were the ratings across relevance classes more balanced. As the survey format did not allow for a selection of indicators based on the quantitative results, the qualitative feedback (free comment fields for each indicator) was used to revise the indicator pool and the framework concept.

#### Revision of the framework concept and indicators (8)

Based on the qualitative feedback from Delphi round 1 the framework concept was revised and additionally more closely aligned with a multidimensional approach to mental health. For this purpose, mental health status was integrated into a ‘staging approach’ [14] with the two dimensions of positive mental health (well-being) and psychopathology [10]. This approach interprets psychopathology not as a categorical but as a dimensional construct, ranging from preclinical symptoms up to manifest mental disorders in varying degrees of severity (called ‘stages’). The staging approach opens up several approaches to strengthening mental health at different levels (promotion: positive mental health, prevention: mental distress, remission: mental disorders, recovery: psychosocial impairment), each related to specific public health measures (health promotion and prevention as well as treatment and rehabilitation).

**Figure 2:**
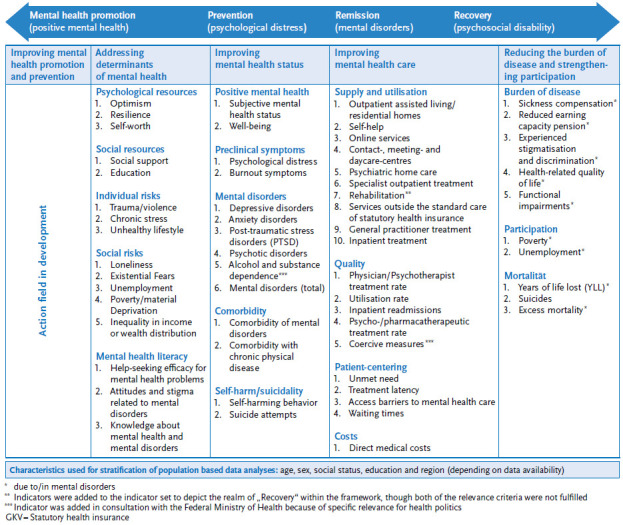
Final framework concept and indicator set of the Mental Health Surveillance Source: Own figure

In order to reflect these conceptual decisions, fields of action were renamed, topics partially differentiated and redistributed (Figure 2): (1) ‘positive mental health’, ‘psychopathology’ and ‘self-harm and suicidality’ were included as characteristics of mental health status in the shared field of action 3 ‘Improving mental health status’. For the gradual mapping of manifestations (staging approach) of mental health problems, the topic of psychopathology was divided into the three topics (1) ‘preclinical symptomatology’; (2) ‘mental disorders’; and (3) ‘comorbidities’. Field of action 5 ‘Reducing the burden of disease and strengthening participation’, which indicates the severity of mental impairments and its consequences, was supplemented by ‘mortality’. (2) ‘Risk factors of mental disorders’ and ‘mental health resources”, which had previously been assigned to two fields of action as they can influence both positive mental health (well-being) and psychopathology, were combined into field of action 2 ‘Addressing determinants of mental health’ and supplemented by ‘mental health literacy’ (formerly the separate field of action 5). (3) In order to reflect the spectrum of public health measures, field of action 1 ‘Improving mental health promotion and prevention’ was added to complement the unchanged field of action 4 ‘Improving mental health care’.

The additional in-depth and Germany-centric research into publications on mental health promotion and prevention made it possible to add twelve further indicators to the indicator pool. However, as these only insufficiently represent the first field of action, they were placed outside the Delphi round 2 evaluation matrix and defined as an area for future development. A first assessment of the relevance of these indicators in Delphi round 2 was conducted to identify possible settings with high relevance for mental health promotion and prevention measures (Annex Table 2).

The socio-demographic characteristics considered potential stratification variables were revised with the help of additional experts at the RKI. ‘Education’ was included as an indicator of social resources. ‘Unemployment’ and ‘poverty’ were classified as indicators of both social risk factors and consequences of disease. In order to homogenise future NCD surveillance, the stratification characteristics of age, gender, social situation, education and region (depending on the respective data availability) [67], on which consensus was previously achieved in the context of Diabetes Surveillance, were adopted.

In the focus groups (6), criteria of public health relevance were discussed with 18 representatives from national stakeholders, which could be used to select specific mental disorders for the MHS in Delphi round 2, including, for example, their incidence, prevalence, disease burden, treatability or also preventability, as well as methodological criteria such as their epidemiological measurability in population-based health studies (e.g. psychometric quality, sparseness of assessment). A proposal for the mental disorders to be selected in Delphi round 2 (Annex Table 2) was accepted by the respondents and slightly modified by splitting the general category ‘stress disorders’ into ‘adjustment disorders’ and ‘post-traumatic stress disorders’.

#### Indicator evaluation Delphi round 2 (online) (9)

In total, 80% (n=16) of the invited stakeholders participated in the first sub-survey, 65% (n=13) in the second sub-survey.

Based on the results, 57 of the 96 indicators from fields of action 2 to 5 were extracted for the final set of indicators (Annex Table 2). A total of 36 indicators were classified as highly relevant and 17 as relevant. Four indicators were included to ensure that, as per definition, each topic should be represented by at least two indicators. Despite its low ranking, the indicator ‘rehabilitation’ was subsequently included within the topic,service provision and service use’ in order to also reflect the area of ‘recovery’ [79], which is anchored in the framework concept.

#### Adoption of the indicator set (10)

The final indicator set was recognised and adopted by the majority of national stakeholders with an approval rate of 85% (response rate: 95%, n=19). One abstention was justified due to the lack of opportunity for a detailed discussion of individual indicators. One rejection was justified by the exclusion of the indicator ‘coercive measures’ as well as criticism of the indicator ‘psycho-pharmacotherapeutic treatment rate’.

#### Expansion of the indicator set (12)

In consultation with the Federal Ministry of Health, two key health policy indicators were added: ‘alcohol and substance dependence’ as a relevant group of mental disorders and ‘coercive measures’ for the area of quality of care. The final set thus contains 60 indicators (Figure 2).

#### Evaluation of the consensus process (11)

Seventeen of the national stakeholders contributed to the evaluation of the consensus process (response rate: 85%) (Annex Table 3). The majority was satisfied with the opportunities to contribute their own opinion (88.2%), found their opinion adequately reflected in the outcomes (82.4%) and rated the procedure as sufficiently transparent (94.1%). In total, 88.2% of respondents felt that the effort required to participate was fit for purpose, 11.6% would have become even more involved if necessary. All respondents would be willing to participate in the further development of MHS in the future.

## 4. Discussion

Following international examples, the RKI has begun to develop a MHS for Germany. The steps taken highlight the increasingly recognised importance of mental health as an aspect of population health and responds to the high demand for an up-to-date and sustainable evidence base for the design of public mental health measures. The MHS aims for more comprehensive and reliable assessments of the mental health of the population by continuously providing data for an indicator set on which consensus has been achieved. The piloting of a German MHS presented in this paper yielded the following results: a structured consensus process condensed the extensive pool of indicators identified by means of a systematic literature search to 60 indicators. These indicators represent a multidimensional framework for public mental health. A broad consensus for the selected set of indicators could be achieved among the involved stakeholders. In order to critically discuss the applied procedure, it will be reflected on the focus of the final indicator set and the strengths and limitations of methodological decisions and next steps to developing the surveillance system will be presented.

## Final indicator set

The final set of indicators is indicative of the different focuses of the stakeholders involved in terms of content. Field of action 1 ‘Improving mental health promotion and prevention’, highlights indicators on settings and measures representing the entire life span. In addition, information on antistigma and awareness campaigns was rated as relevant.

Field of action 2, ‘Addressing determinants of mental health’ looked at personal resources prioritising personality constructs such as optimism, resilience and self-esteem over competencies (communicative, social, and coping-related). Stressors (traumatisation and violence, chronic stress) and health behaviour were rated as central risk factors. Structural factors (poverty and unemployment) were prioritised alongside loneliness as social risk factors. Correspondingly, comparable social resources (education and social support) were selected. For the topic of mental health literacy, which has to date hardly been studied at the population level, none of the three proposed indicators was prioritised.

The special focus in field of action 3 ‘Improving mental health status’ was the prioritisation of mental disorders in the MHS. These disorders include depressive and anxiety disorders representing particularly common diagnostic groups and psychotic disorders as usually severe disorders in terms of course and consequences. In addition, post-traumatic stress disorders were selected, which is closely related to the high prioritisation of traumatisation and violence among the risk factors.

In field of action 4 ‘Improving mental health care’, the entire spectrum of the care landscape received high ratings; community psychiatric services were also included as essential for surveillance. With regard to the issue of quality and patient-centred care, priority was given to indicators that address the entire group of persons with mental disorders requiring treatment (e.g. service use and treatment rates, need for and access to care). Therefore, some indicators with special significance for the specific situation of people with chronic or severe mental disorders (e.g. physical health care and coercive measures) as well as indicators of perceived treatment success and satisfaction from the patients’ point of view were not selected (although the coercive measures indicator was subsequently added, see above).

In field of action 5 ‘Reducing the burden of disease and strengthening participation’, the direct determination of differentiated indicators regarding the individual and societal burden of disease was given preference over summary measures of the burden of disease model or the estimated economic costs. Poverty and unemployment were selected as indicators of participation, which is consistent with the prioritisation of social risk factors in field of action 2. To monitor mortality, overarching indicators of (excess) mortality were given preference over disorder-specific and care-associated measures.

## Lessons learned

Compared to other internationally developed systems, the decision to create a public health-orientated framework has allowed the development of a comparatively comprehensive set of indicators. The framework covers both positive mental health (well-being) as well as a dimensional perspective of psychopathology; it therefore illustrates approaches for public health measures (health promotion, prevention, treatment and rehabilitation) at different levels. Thus, for the future integration of MHS into a superordinate surveillance of non-communicable diseases, consented indicators for many issues are already available. These also cover the association of mental and physical health in terms of common protective and risk factors, reciprocal influencing factors as well as co-morbidity and multimorbidity. A comprehensive surveillance system focused on public health is more capable of reflecting the complexity of health and disease than separate disease- or disorder-focused subsystems. However, this result was only achieved by following the approach taken by the Public Health Agency Canada [24, 76], namely a strict structuring of indicator selection in Delphi round 2 [24, 76] which had already anchored essential elements of the framework concept in the final indicator set. However, the procedure proved to be legitimate, as the stakeholders involved indicated a high level of agreement with the final indicator set despite these specifications.

Selecting indicators based on a systematic search of potential indicators has provided a set of indicators that also contains indicators for which no data suitable for surveillance is available (yet). Unlike data-driven forms of indicator selection [e.g. 27], the process has revealed urgent data needs and research gaps for core areas of public mental health [cf. 23, 24, 80]. The majority of the indicators that were extracted during the extensive literature search came from indicator systems which were already established. In conclusion, a stronger orientation towards existing indicator sets in combination with targeted follow-up research on underrepresented topics or special country needs can be recommended as efficient methods for developing a public mental health surveillance. At the same time, the approach we chose has allowed us to integrate constructs and topics (e.g. mental health literacy) not yet included in established surveillance systems into the structure of a MHS, which reflect more recent developments in monitoring population mental health or that take specific concerns held in Germany into account.

Restricting the indicator search and selection to constructs as opposed to precisely defined operationalisations at numerator and denominator level has also proven to be feasible in other such processes [24, 27, 28] and was indispensable for the feasibility of the consensus-building process. Only this pragmatic simplification has allowed those involved in the selection process to summarise and compare the extraordinarily large number of potential indicators. The development of clear and long-term metadata (title and definition) for the indicators, as well as the choice of their measurement and data basis, only takes place in a second step. This has the advantage, that the current and, as in the context of the COVID-19 pandemic, sometimes rapidly changing landscape of available data can be explored and included in detail. At the same time, research needs can be identified and the use of new inventories or survey methods tested. However, in the course of operationalisation, important decisions regarding content must be made and the methodological quality of the indicators needs to be ensured [8, 81].

The participatory development of an indicator set by a committee of experts and stakeholders is an established procedure [24, 32, 75], but not without alternatives [24, 27, 28, 32, 75]. In this case, a broad acceptance of the result could be ensured, which, in turn, is a prerequisite for the effective use of the surveillance system. It must be recognized, however, that any outcome of collective decision-making processes naturally always depends on the composition of the body involved. For example, differing interests on the committee can lead to the selection or deselection of individual indicators. Although the aim was to win representatives of all relevant stakeholder groups, their respective representation (e.g. of mental health promotion and prevention actors versus service providers of outpatient or inpatient care) can be viewed critically. In the case of the three indicators subsequently added (rehabilitation, coercive measures, alcohol and substance dependence), there is no consensus in the group involved. In the course of the advancement of the MHS in Germany, it seems important not to prioritise these three indicators in the further scientific processing at the expense of those indicators that were determined by consensus.

Main criticisms from the experts and stakeholders involved were doubts about the significance of the ‘psychopharmacotherapeutic treatment rate’ indicator and the demand to include coercive measures. Due to overlaps in content and the difficulty of comparing constructs, the evaluation of indicators was considered difficult. The lack of indicators with reference to occupational health and work and for community psychiatric networking was criticised. In addition, the limited opportunities for an intensive discussion about individual indicators due to the restrictions imposed by the COVID-19 pandemic were deemed regrettable but accepted.

## Outlook

The development of an indicator set represents a first step in the establishment of a MHS in Germany. To establish a sustainable surveillance system for the selected topics, however, the following further work is necessary:

(1) Determining of the data basis available for each indicator: Currently and prospectively available population-based data sources for the quantification of indicators must be explored and evaluated. Concepts to close data gaps must be developed for those indicators that cannot be mapped at present. This includes both the not yet (routine) use of already available data sources as well as the development of new data sources from scratch. For indicators with a considerable need for development, a transitional use of interim indicators should be considered [cf. 23].

(2) Precise definition and operationalisation of indicators: Survey formats or psychometric instruments must be selected, developed and tested for indicators for which data will need to be collected in surveys. To facilitate the use of MHS data as reference values for population norms, preference should be given to open access instruments. In addition, the use of mobile-based digital survey methods for estimating mental health indicators at overall population level should be explored. For indicators based on routine data, appropriate definitions of cases or services to be considered need to be established and suitable data bodies selected. Meanwhile, international comparability must be ensured by giving preference to internationally established measures. At the same time, the operationalisation of indicators must allow for the greatest possible use of routine data and regular primary data collection beyond the RKI’s population studies. Only by doing so a continuous surveillance for the large number of indicators of the MHS can realistically be feasible. If possible, the selected data sources should allow for the defined stratification. Due to the high significance of small-scale results for the planning of measures the factor region plays a particularly important role.

(3) Encouraging continuous data availability: In order to provide close-meshed and continuous data, the regular collection of survey data and use of routine data must be ensured. International experience has shown [82] that this requires a constant influence on the planning of data collections and evaluations in order to place the selected MHS indicators on their agenda.

(4) Dissemination of results: In future, established and innovative formats of health reporting, such as those currently being tested for the Diabetes Surveillance [83], can be used for timely and appropriate MHS reporting. In order to promote their use by the addressees, suitable formats of direct exchange with the relevant actors must be developed.

(5) Integration into NCD surveillance: A prudent selection of the MHS indicators identified here will be integrated into an overarching NCD surveillance. In the long term, a reporting system will be established, which depicts the relevance of mental health for physical health [84], too.

(6) Necessary extensions: Expansion potentials of the MHS indicator set proposed here lie a) in the addition of indicators for the age groups of children and adolescents as well as the elderly in order to map aspects of mental health across the lifespan, and b) in the elaboration of field of action 1 on the basis of the expected progress of prevention reporting [85].

## Conclusion

The piloted development of the present MHS indicator set has proven to be practicable overall. Through the framework concept, a broad public mental health approach could be firmly established and existing gaps in indicator-based reporting in the field of mental health promotion and prevention could be identified. The final set of indicators was selected on the basis of a differentiated assessment by the stakeholders involved. By this means, an essential contribution to the usefulness and acceptance of the system has been made with regard to the quality criteria of a surveillance system for mental health [17]. On this basis the development of the MHS in Germany can be continued. If the next steps are implemented according to plan, the MHS can become a helpful tool to make developments in public mental health in Germany visible in a timely manner, identify needs for interventions and burdened population groups and so contribute to an evidence-based planning and evaluation of public health measures aimed at promoting mental health and reducing the burden of disease of mental disorders.

## Key statements

- Increasingly, mental health is being recognised as a fundamental aspect of population health.
- There is a lack of systematically selected and continuously available data for mental health surveillance in Germany.
- In order to set up a Mental Health Surveillance, the search, definition and selection of indicators for the broad spectrum of mental health was piloted.
- The final set comprises 60 indicators across four fields of action.
- Corresponding indicators for the field of action ‘Mental health promotion and prevention’ remain to be developed.

## Figures and Tables

**Table 1:** Results of indicator evaluation according to field of action and relevance in Delphi round 1 Source: Own table

	Indicators												Sociodemographic stratification characteristics	
	Indicator total		Field of action 1: Promoting mental wellbeing of the population		Field of action 2: Reducing the risks of mental disorders		Field of action 3: Improving mental health care		Field of action 4: Reducing the burden of disease and improving participation		Field of action 5: Strengthening knowledge and acceptance			
Evaluation	Number	%	Number	%	Number	%	Number	%	Number	%	Number	%	Number	%
Highly relevant	61	35.3	1	3.1	17	33.3	21	38.9	15	62.5	7	58.3	8	72.7
Relevant	83	48.0	11	34.4	28	54.9	31	57.4	9	37.5	4	33.3	3	27.3
Medium relevant	27	15.6	18	56.3	6	11.8	2	3.7	0	0.0	1	8.3	0	0.0
Low relevant	2	1.2	2	6.3	0	0.0	0	0.0	0	0.0	0	0.0	0	0.0
**Total**	**173**		**32**		**51**		**54**		**24**		**12**		**11**	

## Appendix

**Annex Table 1:** Participating international experts and national stakeholders Source: Own table The listed persons participated in the development of the indicator set and framework concept (workshop 1, focus groups on the selection of mental disorders, Delphi round 1 or/and Delphi round 2).

Nr.	Name	Institution
1	Dr Marion Aichberger	Department of Psychiatry and Psychotherapy at the Charité Campus Mitte, Charité – Universitätsmedizin Berlin
2	Prof Dr Harald Baumeister	Department of Clinical Psychology and Psychotherapy, Institute of Psychology and Education, University of Ulm
3	Prof Dr Anke Bramesfeld	Ministry for Social Affairs, Health and Equal Opportunities of Lower Saxony; Institute for Epidemiology, Social Medicine and Health System Research, Hannover Medical School (MHH)
4	Dr Daniel Hugh Chisholm	World Health Organization, Regional Office for Europe
5	Jurand Daszkowski	Federal Association of Psychiatry Experienced (BPE)
6	Prof Dr Freia de Bock	Federal Centre for Health Education (BZgA)
7	Dr Julian Dilling	National Association of Statutory Health Insurance Funds
8	Dr Theresa Eichhorn	Federal Chamber of Psychotherapists in Germany (BPtK)
9	Prof Dr Wolfgang Gaebel	WHO Collaborating Centre DEU-131; Rhineland Regional Council (LVR) – Klinikum Düsseldorf, Kliniken der Heinrich-Heine-Universität Düsseldorf
10	Prof Dr Dr Martin Härter	University Medical Center Hamburg-Eppendorf, Center for Psychosocial Medicine, Department of Medical Psychology; German Network Health Services Research (DNVF)
11	Prof Dr Dr Andreas Heinz	Department of Psychiatry and Psychotherapy at the Charité Campus Mitte, Charité – Universitätsmedizin Berlin
12	Emily Hewlett	Organization for Economic Cooperation and Development (OECD)
13	Prof Dr Frank Jacobi	Department of Clinical Psychology and Psychotherapy, Psychologische Hochschule Berlin
14	Dr Alessa Jansen	Federal Chamber of Psychotherapists in Germany (BPtK)
15	Dr Joseph Kuhn	Bavarian Health and Food Safety Authority (LGL)
16	Prof Dr Jutta Lindert	University of Applied Sciences Emden/Leer; European Public Health Association, Section Public Mental Health
17	Prof Dr Jürgen Margraf	Mental Health Research and Treatment Center, Ruhr-University Bochum
18	Alexandra Matzke	German Depression League e.V.
19	Dr Hanne Melchior	National Association of Statutory Health Insurance Physicians (KBV)
20	Prof Dr Andreas Meyer-Lindenberg	Central Institute of Mental Health, Medical Faculty Mannheim, Universität Heidelberg; German Association for Psychiatry, Psychotherapy and Psychosomatics e.V. (DGPPN)
21	Dr Dietrich Munz	Federal Chamber of Psychotherapists in Germany (BPtK)
22	Dr Angelika Nebe	Federation of German Pension Insurance Institutions (DRV Bund)
23	Dr Heather Orpana	Public Health Agency Canada (PHAC)
24	Dr Judith Peth	University Medical Center Hamburg-Eppendorf, Center for Psychosocial Medicine, Department of Medical Psychology
25	Prof Dr Ulrich Reininghaus	Department of Public Mental Health, Central Institute of Mental Health, Medical Faculty Mannheim, Universität Heidelberg
26	Prof Dr Steffi Riedel-Heller	Institute of Social Medicine, Occupational Health and Public Health (ISAP), Faculty of Medicine, University of Leipzig; German Association for Psychiatry, Psychotherapy and Psychosomatics e.V.
27	Dr Uwe Rose	Federal Institute for Occupational Safety and Health (BAuA)
28	Dr Ursula von Rüden	Federal Centre for Health Education (BZgA)
29	Prof Dr Georg Schomerus	Department of Psychiatry and Psychotherapy, University of Leipzig Medical Center (ULMC), Medical Faculty, University of Leipzig
30	Daniela Schuler	Swiss Health Observatory (Obsan)
31	Prof Dr Martin Schütte	Federal Institute for Occupational Safety and Health (BAuA)
32	Dr Thomas Stracke	Federal Ministry of Health (BMG)
33	Thomas Voigt	German Depression League e.V.

GKV = Statutory health insurance, WHO = World Health Organization

**Annex Table 2:** Results of indicator assessment in Delphi Round 2 Source: Own table

	n	Ranking (cumulative)^1^	Consensus^2^
**Field of action 1: Improving mental health promotion and prevention** (under development; assessment outside Delphi process for initial assessment of the field of action)			
**Topic: Settings for possible indicators**			
Work environment/company	13	38	85%
Unemployment	13	43	62%
Kindergarten/daycare centre (KiTa)	13	44	77%
Family	13	59	46%
Nursing/care facility (senior citizens, people with disabilities)	13	66	54%
Municipality/community/district	13	67	38%
University/college/training company/vocational school	13	69	38%
School	13	82	0%
**Topic: Indicators with currently available data**			
Anti-stigma and awareness raising	13	66	69%
SHI-supported measures in daycare centres for promotion and prevention in the field of mental health	13	71	77%
SHI-supported measures in schools for promotion and prevention in the field of mental health	13	74	77%
Relaxation or stress management offers by the employer	13	75	46%
Early help	13	82	62%
Health promotion measures at the workplace	13	85	38%
Stress management measures at the workplace	13	88	31%
Certified prevention services in the field of mental health	13	88	46%
Employer’s measures to prevent psychosocial risk factors in the workplace	13	92	31%
Risk assessment of mental health at the workplace	13	96	46%
Measures to deal with psychosocial risk factors at the workplace	13	98	46%
Measures to strengthen psychosocial health in care facilities	13	99	31%
**Topic: Indicators for the self-report**			
Use	13	28	54%
Offer	13	29	69%
Demand	13	31	46%
Quality	13	42	31%
**Field of action 2: Addressing determinants of mental health**			
**Topic: Psychological resources**			
Optimism	16	44	75%
Resilience	16	46	69%
Self-efficacy	16	63	44%
Coping skills	16	64	31%
Social/communicative competences	16	65	31%
Spirituality	16	107	6%
**Topic: Social resources**			
Social support	16	37	56%
Education	16	38	63%
Life Domain Balance/work Life Balance	16	47	44%
Social and political participation	16	56	19%
Access to recreational and leisure opportunities	16	62	19%
**Topic: Individual risks**			
Trauma/violence	16	42	69%
Chronic stress	16	55	50%
Unhealthy lifestyle	16	63	44%
Burden of chronic illness and/or chronic pain	16	64	44%
Experience of discrimination	16	67	31%
Stressful childhood experiences	16	69	38%
Exposure to family members with mental health problems	16	88	25%
**Topic: Social risks**			
Loneliness	16	69	56%
Existential fears	16	70	56%
Unemployment	16	72	44%
Poverty/material deprivation	16	74	63%
Inequality in income or wealth distribution	16	78	56%
Homelessness	16	84	38%
Precarious housing conditions	16	87	38%
Stressful living environment	16	93	25%
Stressful working conditions	16	93	25%
**Topic: Mental health literacy (Gesundheitskompetenz)**			
Help-seeking efficacy for mental health problems	13	24	77%
Attitudes and stigma related to mental disorders	13	26	69%
Knowledge about mental health and mental disorders	13	28	54%
**Field of action 3: Improving mental health status**			
**Topic: Positive mental health**			
Subjective mental health status	15	22	53%
Well-being	15	23	47%
**Topic: Preclinical symptoms**			
Psychological distress	15	18	80%
Burnout symptoms	15	27	20%
**Topic: Mental disorders**			
Depressive disorders	14	47	79%
Anxiety disorders	14	58	71%
Post-traumatic stress disorders (PTSD)	14	60	64%
Psychotic disorders	14	67	36%
Personality disorders	14	68	50%
Severe mental disorders	14	73	36%
Alcohol and substance dependence^**^	14	85	21%
Somatoform disorders	14	86	14%
Adjustment disorders	14	86	29%
**Topic: Comorbidity**			
Comorbidity of mental disorders	14	20	57%
Comorbidity with chronic physical diseases	14	22	43%
**Topic: Self-harm/suicidality**			
Self-harming behaviour	14	23	93%
Suicide attempts	14	25	79%
Suicidal thoughts and/or plans	14	36	29%
**Field of action 4: Improving mental health care**			
**Topic: Supply and utilisation**			
Outpatient assisted living/ residential homes	13	53	77%
Self-help	13	75	46%
Online services (self-help, counselling, therapy)	13	77	54%
Contact-, meeting- and daycare-centres	13	79	54%
Psychiatric home care	13	79	54%
Specialist outpatient treatment	13	85	62%
Rehabilitation^*^	13	86	38%
Services outside the standard care of statutory health insurance	13	88	54%
General practitioner treatment (primary psychosomatic health care)	13	94	54%
Social psychiatric care	13	95	31%
Inpatient treatment	13	97	54%
Crisis services and counselling centres	13	106	23%
**Topic: Quality**			
Physician/psychotherapist treatment rate (among patients with documented diagnosis of mental disorders)	13	50	77%
Utilisation rate (in population with mental disorders)	13	50	69%
Inpatient readmissions	13	51	62%
Psycho-/pharmacotherapeutic treatment rate (among patients with documented diagnosis of mental disorders)	13	53	62%
Treatment continuity after inpatient stay	13	59	46%
Psychiatric emergencies	13	66	38%
Somatic health care for people with mental disorders	13	78	31%
Quality target achievement in the Disease Management Programme (DMP) Depression	13	87	8%
Coercive measures^**^	13	91	8%
**Topic: Patient-centering**			
Unmet need	13	37	54%
Treatment latency	13	41	54%
Access barriers to mental health care	13	46	46%
Waiting times	13	47	54%
Perceived treatment success (patient-reported outcome)	13	50	46%
Perceived patient orientation (patient-reported experience)	13	52	46%
**Topic: Costs**			
Direct medical costs^***^ (not voted upon)			

**Field of action 5: Reducing the burden of disease and strengthening participation**			
**Topic: Burden of disease**			
Sickness compensation due to mental disorders	14	51	57%
Reduced earning capacity pension due to mental disorders	14	53	64%
Experienced stigmatisation and discrimination due to mental disorders	14	58	57%
Health-related quality of life in mental disorders	14	59	57%
Functional impairments due to mental health reasons	14	62	57%
Disability to work due to mental disorders	14	64	50%
Measures of the Burden of Disease Model for disease burden (DALY, YLD)	14	70	36%
Economic costs due to mental disorders	14	87	21%
**Topic: Participation**			
Poverty among people with mental disorders	14	33	57%
Unemployment among people with mental disorders	14	35	57%
Reintegration into labour market of people with mental disorders	14	39	50%
Social and political participation of people with mental disorders	14	47	21%
Homelessness of people with mental disorders	14	56	14%
**Topic: Mortality**			
Measure of the Burden of Disease Model for mortality (YLL)	14	33	71%
Suicides	14	36	79%
Excess mortality of mental disorders	14	48	50%
Alcohol related deaths	14	56	36%
Drug related deaths	14	58	36%%
Suicides during or after inpatient psychiatric treatment	14	63	29%

**Notes to Annex Table 2:**

Blue bold = highly relevant indicator (criteria rank AND consensus met), included in final indicator set

Black font = relevant indicator (criteria rank OR consensus met), included in final indicator set

Light grey font = not relevant indicator (no relevance criterion met), not included in the final indicator set

Blue background = indicator ranked in the top 50% of the indicators of a topic (odd number rounded down)

Grey shaded = indicator that more than 50% of the respondents ranked in the top 50% of the indicators of a thematic field = number of ratings given by the participating stakeholders

^1^ Ranking (cumulative) = sum of the ranks of an indicator within a topic

^2^ Consensus = Percentage of participating stakeholders who placed the indicator in the top 50% of indicators for a topic (rounded down if odd number)

^*^ Indicator was included in the indicator set in order to depict the area of ‘recovery’ within the framework model, although neither of the two relevance criteria was met.

^**^ Indicator was subsequently included in the indicator set in consultation with the Federal Ministry of Health due to its relevance to health policy.

^***^ Indicator was included in the indicator set without voting because it was the only one representing the topic.

GKV = Statutory health insurance, ASHIP = National Association of Statutory Health Insurance Physicians, DALY = Disability-adjusted life years,

YLD = Years lived with disability, YLL = Years of life lost, SHI = statutory health insurance

**Annex Table 3:** Results of the evaluation of the consensus process Source: Own table

	**„Rather agree“ or „Agree“**		**„Rather disagree“ or „Disagree“**			
	**n**	**%**	**n**	**%**		
1. I had sufficient opportunity to express my opinion in the course of the consensus process on the development of an indicator set.	15	88.2	2	11.8		
2. I find my opinion sufficiently reflected in the results.	14	82.4	3	17.6		
3. I found the procedure for selecting the core indicators sufficiently transparent (e.g. regarding the steps of the consensus process, evaluation criteria, documentation of results).	16	94.1	1	5.9		
	**„I would have been more involved if needed.“**		**„The effort was reasonable.“**		**„The effort was too high.“**	
	**n**	**%**	**n**	**%**	**n**	**%**
4. How do you rate the effort required for your participation in the consensus process in relation to its purpose?	2	11.6	15	88.2	0	0
	**„yes“**		**„no“**			
	**n**	**%**	**n**	**%**		
5. Would you be willing to participate in the development of the Mental Health Surveillance in the future?	17	100	0	0		

n = Number of ratings given by the participating stakeholders, assessment of questions 1 to 3 on a four-point scale
